# Determinants of uptake of first dose of intermittent preventive treatment among pregnant women in a secondary health Centre in Maiduguri, Nigeria

**DOI:** 10.1186/s12884-020-03388-8

**Published:** 2020-11-25

**Authors:** Ahmed Dahiru Balami, Salmiah Md. Said, Nor Afiah Mohd. Zulkefli, Norsa’adah Bachok, Bala Audu

**Affiliations:** 1 Department of Community Health, Faculty of Medicine and Health Sciences, Universiti Putra Malaysia, UPM, 43400 Serdang, Selangor Malaysia; 2 Unit of Biostatistics and Research Methodology, School of Medical Sciences , Universiti Sains Malaysia , Kubang Kerian , 15200 Kota Bharu , Kelantan Malaysia; 3 Department of Obstetrics and Gynaecology, University of Maiduguri, PMB 1069 Maiduguri, Borno State Nigeria

**Keywords:** Intermittent preventive treatment, Pregnant women, Malaria, Nigeria

## Abstract

**Background:**

Studies on uptake of first dose of intermittent preventive treatment in pregnancy (IPTp) are lacking, despite it being a predictor of subsequent doses. This study aimed at assessing the determinants of uptake of first dose of IPTp among pregnant women at the State Specialist Hospital, Maiduguri.

**Methods:**

A cross-sectional study was conducted, in which respondents were selected using a systematic random sampling method, and structured questionnaires were used to obtain information from them. Chi-squared test was used to determine factors associated with uptake of first IPTp dose, while a further multivariate logistic regression was performed to determine its predictors.

**Results:**

Three hundred and eighty respondents answered the survey, whose ages ranged from 15 to 45 years, and 86.8% were multigravid. Sixty five percent of them were aware of IPTp, and 34.7% believed that IPTp could be harmful to their pregnancies. Over a half of the respondents (52.9%) believed that taking all their IPTp medicines was very good for their pregnancies, while 45.0% felt that taking their IPTp medicines was very pleasant. Only two respondents (0.5%) stated that it was very untrue that their significant others thought that they should take all their IPTp medicines. Half of the respondents said it was very easy for them to take all their IPTp medicines even if they were experiencing mild discomforts while taking them. Less than a half (42.37%) had received their first dose of IPTp. In bivariate as well as multivariate analysis, only higher level of knowledge was significantly associated with uptake of first IPTp dose. Those with better knowledge of IPTp were about twice more likely to have taken their first dose of IPTp, compared to those with lower knowledge of IPTp (AOR = 1.85; 95% CI: 1.17–2.92).

**Conclusions:**

Knowledge of IPTp as well as its uptake, were sub-optimal in this study. Since knowledge of IPTp significantly predicts uptake of the first dose of IPTp, there is the need to implement health education campaigns to raise the awareness of pregnant women and their families on the need to receive and comply with it.

**Supplementary Information:**

The online version contains supplementary material available at 10.1186/s12884-020-03388-8.

## Background

Malaria infection during pregnancy could result in adverse consequences like miscarriage [1, 2], anaemia [3, 4], pre-term delivery [5, 6], stillbirth [7, 8], and low birth weight [9, 10]. High prevalence of malaria infection has been reported among antenatal care attendees in different health centres across Nigeria (South-eastern Nigeria) [11–19]. In Borno state, Nigeria, the prevalence of malaria among pregnant women was 48.1% at the state’s largest secondary health centre [20]; 60.3% in its only Teaching Hospital [21] and 44.5% three years later, at the same Teaching Hospital [22]. A systematic review of four trials revealed that receiving two doses of intermittent preventive treatment in pregnancy (IPTp) with Sulphadoxine-Pyrimethamine (SP) reduced the risk of having placental malaria, anaemia, and low birth weight [23]. Another systematic review of 14 cluster-randomized and eight individually-randomized controlled trials had also revealed the protective efficacy of IPTp-SP in reducing low birth weight [24]. As such, the World Health Organization (WHO) as well as the Federal Ministry of Health (FMOH) such, recommends that pregnant women in sub-Saharan Africa receive at least three doses of IPTp-SP during their pregnancies [25, 26]. Each dose is to comprise of three tablets (500 mg Sulphadoxine and 25 mg Pyrimethamine per tablet) at scheduled antenatal care visits. The first dose is to be given at the beginning of the second trimester, while subsequent doses should be given at least one month apart. It can be given on an empty stomach, or with food; should be directly observed by a health worker; should not be taken concomitantly with daily Folic acid supplementation; and should not be given to those receiving Co-trimoxazole prophylaxis [27]. Despite the recommendations by the WHO and FMOH, the Nigerian Health and Demographic Survey revealed that only 13.9% of pregnant women in Borno State had received any single dose of IPTp-SP during their pregnancies, 6.7% had received 2 doses, while 1.9% had received 3 doses [28].

Receipt of the first dose of IPTp seems to be very crucial, as timely uptake of first dose was significantly associated with taking the full recommended doses [29]. Also, receipt of first dose of IPTp at four to six months of gestation had been shown to significantly predict receiving optimal doses of IPTp [30]. Although studies on the determinants of uptake of first IPTp dose are lacking, it may be logical to assume that its predictors overlap with those of full dose uptake. For example, early onset of antenatal visits was significantly associated with uptake of the first dose [31], as well as full dose of IPTp [32, 33]. Availability of SP at the health facility also predicted first dose (Protas et al., 2016) [31] and full dose [34] uptake. Socio-demographic factors like advanced maternal age [35], higher educational attainment [36–39], employment [40] and urban residence [41] have been shown to predict higher IPTp uptake. In Blantyre, Malawi, multigravidae with three pregnancies and above were more likely to have not received the recommended IPTp regimen [42]. A systematic review identified education, knowledge about malaria, socio-economic status, and parity, as major determinants of IPTp coverage in sub-Saharan Africa [43].

Even though psychological factors like motivation and self-efficacy have been reported to be important determinants of health behaviour [44, 45], knowledge of IPTp seems to be the only psychological factor whose association with IPTp uptake has been studied, and this was found to be significant [46–48]. While motivation comprises personal attitudes as well as the subjective social norms toward a particular health behaviour [44], self-efficacy refers to a person’s perceived ability to perform the behaviour [49]. Understanding the role played by these factors (motivation and self-efficacy) would add to the present knowledge of determinants of uptake of first IPTp dose. This would guide the development of health education modules and other intervention programmes to ultimately improve IPTp uptake. As such, this study aimed at assessing the determinants of uptake of first dose of IPTp among pregnant women at the State Specialist Hospital, Maiduguri.

## Methods

### Study area

The study area was Maiduguri, the capital of Borno state, in northern Nigeria. Maiduguri has a population of 282,409 males and 257,607 females [50], with farming and trading as the main economic activities [51]. Thirty nine percent of pregnant women in Borno state receive antenatal care from a skilled provider [28]. Maiduguri experiences between 30,605 and 43,860 cases of malaria in pregnancy annually [52]. The study location was the ante-natal care clinic of the State Specialist Hospital, Maiduguri, which is the largest of the three secondary health centres in Maiduguri. The hospital has four clinical departments thus: internal medicine, surgery, paediatrics and obstetrics and gynaecology. The ante-natal care (ANC) clinic is run as a sub-unit of the obstetrics and gynaecology department. It is run from Monday through Friday, with an average of about 100 clients per clinic day. Mondays are reserved for first-time ANC attendees, while the other four days are for follow-up visits. Pregnant women coming to the clinic early in the morning for their ANC are first made to register and get an ANC card from the medical record’s office. After obtaining their card, they get seated serially in the waiting area. Once registration has been closed, they are collectively given health talks by the midwives before the antenatal consultations start. During the consultations, SP is appropriately prescribed for the pregnant women to buy at the pharmacy, and to take when they reach home. Studies in some centres in Nigeria have shown the average gestational age at first antenatal visit to be around four months [53, 54].

### Recruitment of respondents

A cross-sectional study design was used for this study, with respondents recruited from eight consecutive Monday clinics, from 30 January to 13 March, 2017. The one-proportion formula, Z_1-α/2_^2^ p (1-p)/d^2^ [55] was used to calculate the sample size for this study. Substituting 1.96 and 0.05 for ‘Z_1-α/2_’ and ‘d’ respectively, and reported IPTp uptake in Maiduguri (0.139) [28] as ‘p’, this gave a minimum required sample size of 184 participants. Fluency in Hausa (based on self-reporting), as well as being at their first antenatal care visit for the index pregnancy, were the criteria for eligibility for this study. Since the study also served as baseline for a prospective study, and therefore excluded those with pregnancies above five months as well as those with hypertension or diabetes mellitus, as they could affect the pregnancy outcomes [56, 57].

For each day of recruitment, a complete list of the attendees was obtained before the health talks get started, and those with any condition in the exclusion were screened out. The resultant list then served as the sampling frame for that day. Participants were then selected using a systematic random sampling method with 2 as the k^th^ element. The first respondent was selected from the first two eligible attendees in the list, using the table of random numbers, and subsequent respondents were then selected by consecutively skipping one eligible respondent to select the next one, until the last eligible attendee.

### Variables

Having taken the first IPTp dose was the dependent variable. The independent variables were: respondents’ characteristics, knowledge, motivation and self-efficacy toward IPTp.

### Study instrument and data collection

Structured questionnaires (Additional file 1) comprising of five sections were used to collect data from the respondents. Section one was on participants’ characteristics, which had a total of nine questions. Section two assessed participants’ knowledge of IPTp-SP, with a set of eight questions, each having three options, ‘Yes’, ‘No’ and ‘I don’t know’. A correct answer was scored one (1) point, while an incorrect answer or ‘I don’t know’ were scored zero (0). Section three assessed motivation, using two sets of questions: one set consisting of four questions on a Likert scale of five (scored from 1 to 5 points), while the other consisted of two questions, both on Likert scales of six (scored from 1 to 6 points). Section four assessed self-efficacy, with two questions on a Likert scale of four (scored from 1 to 4 points). Section five asked whether or not the respondents had taken any IPTp-SP.

The questionnaire was first developed in English language and then forwardly translated into Hausa language by a senior university academic staff of the linguistics department, following which the Hausa version was backwardly translated to English language by a different person of similar qualification. The original English version and the backward English translation were then compared by a public health specialist who was not part of the researchers. The Hausa version was then pre-tested on 190 respondents. The internal consistency was measured for sections 3 and 4, since there items were on scales, and the Cronbach’s alpha scores were 0.835 and 0.793 respectively, which was considered acceptable [58]. A re-test with the same questionnaire was then given to 50 out of the initial 190 respondents two weeks later to test for reliability, and none of Cohen’s kappa values were less than 0.6 for the items of the knowledge section [59], while the intra-class correlation coefficients were above 0.7 for all items of the motivation and self-efficacy sections. Face-to-face interviews with the Hausa questionnaires were used to obtain information from the respondents. Five enumerators who were all fluent in Hausa language and had diplomas in Community Health were engaged.

### Data analyses

The raw data obtained from the study is attached to this article as a spreadsheet file (Additional file 2). Data analysis was done using IBM SPSS version 22. Income level was categorized into those without any income, those who earned below, and those who earned at or above the Nigerian minimum wage (N18,000 per month). The total scores for knowledge, motivation and self-efficacy were obtained, and then categorised based on the cut-off point from a previous study [59], thus: ‘high’, if they were 70% or above of the total obtainable scores, and ‘low’, if they were less than 70% of the total obtainable scores for that variable. Frequency and percentage were used to summarise the categorical data. Chi squared test was performed to determine the association between IPTp uptake and the factors studied, after which factors with level of significance below 0.25 were included in a multivariate logistic regression to obtain the predictors of IPTp uptake.

### Ethical concerns

Ethical clearance was obtained from the Ethics Committee of the State Specialist Hospital (SSH/GEN/64/Vol.1) and Ethics Committee for Research Involving Human Subjects of the Universiti Putra Malaysia (UPM) (UPM/TNCPI/RMC/1.4.18.2). Informed consent was also obtained from each respondent verbally, after they had been taken through the respondent information sheet. They were informed that their consent would be required to participate in a study in which they would be asked questions on their knowledge, attitude and practice regarding some aspects of malaria prevention. Even though they were highly encouraged to participate by the enumerators, it was made clear to them that participation was voluntary, and they may decide not to respond to certain questions without explanation. They were assured that their responses would remain anonymous, confidential, and strictly for research purposes. No additional consent was obtained from the husbands or guardians of those below 18 years, as they were considered emancipated minors, since they were married women.

## Results

**Fig. 1:**
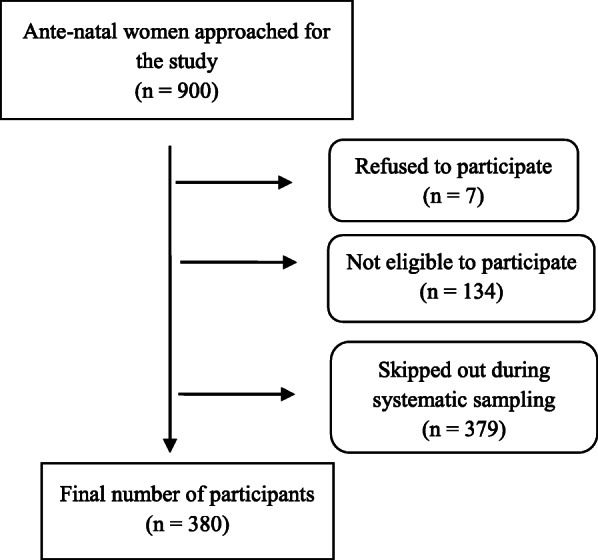
Flow chart of recruitment of respondents

As shown in the flow chart in Fig. 1, a total of 900 antenatal women had been approached to participate in the study, seven of whom declined without giving any reason, except not being interested; 134 were not eligible; while 379 were skipped in the systematic sampling process. This resulted in 380 respondents participating in the study, whose ages ranged from 15 to 45 years, with mean (SD) age of 26.5 (5.8) years. Respondents’ characteristics are presented in Table 1. Most of them were married in a monogamous setting (77.1%). Over half (54.7%) were unemployed, with only 7.6% earning up to the minimum wage as their monthly income. Of the respondents, 13.2% were primigravid, and almost a third (27.1%) had history of a previous miscarriage.

**Table 1:** Respondents’ characteristics

Variable	n	%
**Age group**		
Less than 20	33	8.7
20 years and above	347	91.3
**Family type**		
Widowed	2	0.5
Monogamous	293	77.1
Polygamous	85	22.4
**Occupational status**		
Employed	168	44.2
Not employed	212	55.8
**Income level**		
None	211	55.5
Below minimum wage	140	36.8
At and above minimum wage	29	7.6
**Type of residence**		
Permanent resident	279	73.4
Internally displaced	101	26.6
**Gravidity**		
Primigravid	50	13.2
Multigravida	195	51.3
Grand multigravida	135	35.5
**Previous miscarriage**		
Yes	103	27.1
No	277	72.9

Table 2 shows the respondents’ responses to the various questions on IPTp. Less than half of them (42.1%) correctly identified Fansidar (Sulphadoxine-pyrimethamine) as the drug used for IPTp, and a large number of them (41.8%) believed that IPTp could be harmful to their pregnancies.

**Table 2:** Respondents’ knowledge of IPTp

Question	Response					
	I don’t know		Wrong		Correct	
	n	(%)	n	(%)	n	(%)
Are you aware of the medicine given during pregnancy for protection against malaria?	38	(10.0)	95	(25.0)	247	(65.0)
Chloroquine is the medicine given for IPTp	102	(26.8)	115	(30.3)	163	(42.9)
Fansidar is the medicine given for IPTp	117	(30.8)	103	(27.1)	160	(42.1)
2 tablets of the medicine are given during IPTp	119	(31.3)	78	(20.5)	183	(48.2)
3 tablets of the medicine are given during IPTp	115	(30.3)	88	(23.2)	177	(46.6)
4 tablets of the medicine are given during IPTp	135	(35.5)	48	(12.6)	197	(51.8)
The medicine given to pregnant women for IPTp can be harmful to the pregnancy	62	(16.3)	159	(41.8)	159	(41.8)
Can IPTp be taken on an empty stomach?	66	(17.4)	182	(47.9)	132	(34.7)

As shown in Table 3, over a half of the respondents believed that taking all their IPTp medicines was very good for their pregnancies, (52.9%) while the others had some reservations about them, though to varying degrees. Less than a half however felt that taking their IPTp medicines was very pleasant (45.0%). As presented in Table 4, two respondents (0.5%) had stated that it was very untrue that their significant others thought that they should take all their IPTp medicines, but 27.4% said it was very true that their significant others thought that they should take all their IPTp medicines even when they were not feeling sick.

**Table 3:** Respondents’ level of personal motivation for taking IPTp

STATEMENT	Response				
For the remaining duration of your pregnancy, how good or bad would it be for your health … .	Very bad	Somewhat bad	Neither bad nor good	Somewhat good	very good
To take all the medicines given to you for preventive treatment of malaria in pregnancy?	3 (0.8)	5 (1.3)	29 (7.6)	142 (37.4)	201 (52.9)
Take all the medicines given to you for preventive treatment of malaria in pregnancy even when you don’t feel sick	2 (0.5)	9 (2.4)	26 (6.8)	153 (40.3)	190 (50.5)
**For the remaining duration of your pregnancy, how pleasant or unpleasant would it be for you …**	**very unpleasant**	**Somewhat pleasant**	**neither unpleasant nor pleasant**	**Somewhat pleasant**	**very pleasant**
To take all the medicines given to you for preventive treatment of malaria in pregnancy?	5 (1.3)	9 (2.4)	29 (7.6)	166 (43.7)	171 (45.0)
To take all the medicines given to you for preventive treatment of malaria in pregnancy even when you don’t feel sick	4 (1.1)	11 (2.9)	30 (7.9)	169 (44.5)	166 (43.7)

**Table 4:** Respondents’ level of motivation for taking IPTp: social support

STATEMENT	Response					
Please tell us how true or untrue it is for you	very untrue	mostly untrue	untrue	true	mostly true	very true
Most people who are important to you think you should …						
Take all the medicines given to you for preventive treatment of malaria in pregnancy?	2 (0.5)	6 (1.6)	20 (5.3)	143 (37.6)	111 (29.2)	98 (25.8)
Take all the medicines given to you for preventive treatment of malaria in pregnancy even when you don’t feel sick	2 (0.5)	4 (1.1)	25 (6.6)	143 (37.6)	102 (26.8)	104 (27.4)

The respondents’ self-efficacy for taking IPTp is presented in Table 5. While four respondents (1.1%) said it was very hard for them to take all their IPTp medicines, around a half (49.5%) said it was very easy for them to take all their IPTp medicines even if they were experiencing mild discomforts while taking them. Less than a half (42.37%) had received their first dose of IPTp.

**Table 5:** Respondents’ level of self-efficacy for taking IPTp

STATEMENT	Response			
Right now, how easy or hard would it be for you to …	Very hard	Hard	Easy	Very easy
Take all the medicines given to you for prevention against malaria during pregnancy?	4 (1.1)	15 (3.9)	182 (47.9)	179 (47.1)
Take all the medicines given to you for prevention against malaria during pregnancy even when you experience mild discomfort taking them?	2 (0.5)	19 (5.0)	171 (45.0)	188 (49.5)

Table 6 shows the association between uptake of first IPTp dose and other factors studied. Level of knowledge was the only factor significantly associated with uptake of first IPTp dose (*χ*^2^ = 7.04, *df* = 1, *p* = 0.008).

**Table 6:** Association of study variables with uptake of first IPTp dose

Variables	Uptake of first IPTp dose				***χ***^***2***^	***df***	***p***
	Yes (***n*** = 247) ***n*** (%)		No (***n*** = 133) ***n*** (%)				
**Age group**					0.00	1	0.995
Less than 20	19	(8.7)	14	(8.7)			
20 years and above	200	(91.3)	147	(91.3)			
**Residence**					0.43	1	0.512
Permanent	158	(72.1)	121	(75.2)			
IDP	61	(27.9)	40	(24.8)			
**Income**					1.85	2	0.396
None	128	(58.4)	83	(51.6)			
Below minimum wage	76	(34.7)	64	(39.8)			
At and above minimum wage	15	(68)	14	(8.7)			
**Gravidity**					0.969	2	0.616
Primigravida	32	(14.6)	18	(11.2)			
Multigravida	110	(50.2)	85	(52.8)			
Grandmultigravida	77	(35.2)	58	(36.0)			
**Miscarriage**					1.57	1	0.211
Yes	54	(24.7)	49	(30.4)			
No	165	(75.3)	112	(69.6)			
**Knowledge**					7.04	1	0.008
Low	171	(78.1)	106	(65.8)			
High	48	(21.9)	55	(34.2)			
**Motivation**					0.15	1	0.703
Low	23	(10.5)	15	(9.3)			
High	196	(89.5)	146	(90.7)			
**Self-efficacy**					0.45	1	0.503
Low	15	(6.8)	14	(8.7)			
High	204	(93.2)	147	(91.3)			

In multivariate logistic regression, the model fit, as Hosmer Lemeshow significance value was 0.599. The Negelkerke’s R square, showed that the model explained only 3% variation in uptake of first IPTp dose. As presented in Table 7, having higher knowledge of IPTp was associated with about twice the odds of taking first IPTp dose, compared to those with lower knowledge of IPTp (Adjusted Odds Ratio (AOR) = 1.86; 95% CI: 1.18–2.94, *p* = 0.008).

**Table 7:** Determinants of uptake of first IPTp dose

Factors	B	SE	Wald	***df***	***p***	Adjusted OR	95% CI
**Knowledge**							
Low						1	
High	0.62	0.23	7.06	1	0.008	1.86	1.18–2.94
**Miscarriage**							
Yes						1	
No	−0.30	0.23	1.68	1	0.195	0.74	0.47–1.17

## Discussion

Overall, only around a half of the respondents answered each of the questions on IPTp correctly. Responses for the motivation and self-efficacy sections were better, as less than one tenth gave a negative response to each of their items. Just over 40 % of the respondents in this study had taken their first dose of IPTp. Compared to a previous study in the south-southern part of Nigeria (41.3%) [60], a higher proportion in this study was aware of IPTp (65.0%). A higher level of awareness for IPTp (84.4%) had however been reported in a different study among ante-natal care attendees in the Federal Capital City, Abuja [61]. In a rural community in the south-west, a lesser proportion of them were aware of IPTp (65.0% versus 67.0%), while a greater proportion knew that Fansidar (SP) was the recommended drug for IPTp (67.0% versus 64.8%) [62]. This discrepancy points to the possible role of educational level and place of residence in influencing knowledge of IPTp. Health education is more likely to be intensified at secondary-level health centres (like the study location for this research) compared to Primary Health Care centres in the rural areas, which could explain the greater awareness of IPTp among the group in this study. However, since some level of education is likely to enable one identify the name of the medicine she received, the higher proportion of persons with some education among the rural population (92.3% versus 58.9%), was probably why more of them were able to correctly identify SP as the recommended drug for IPTp. It was also noted in this study that even though only 160 participants correctly identified SP as the drug for IPTp, a higher number (177) correctly stated that three tablets were given. This should be expected, as not all those who can recognise a drug may be able to identify its name. The general belief that medicines should be taken only after one has eaten, was probably the reason why only a few (34.7%) mentioned that SP for IPTp could be taken even on an empty stomach.

In the study location, SP is prescribed to antenatal care attendees by the health workers at the health facility. This could explain the positive attitudes of the women towards it, as only a negligibly small number felt it was either very bad or somewhat bad to their health, with most respondents believing that it was either very good or somewhat good. This highlights the possibility of great level of trust by the women for the health workers, as they seemed comfortable about the safety of the medicines their health workers had given to them. Only a negligibly low percent of the respondents stated that taking IPTp was either very unpleasant or somewhat unpleasant, which is not surprising, considering the general acceptability of SP among pregnant women in Nigeria, and the very few reported side effects, most of which are transient and tolerable [26] . Considering the culture in northern Nigeria where women most at times need to obtain permission from their husbands before leaving the house, even for antenatal care [63], a husband who would allow his apparently healthy wife to go to the hospital for antenatal care is likely to be a very supportive one, which could have accounted for the high social support for taking IPTp from the respondents’ significant others.

Uptake of first dose of IPTp in this study was better than the overall uptake of first IPTp dose in Borno state (13.9%) [28] which was probably because the national survey drew its respondents from the community, of which not all had necessarily commenced antenatal care. Also in this study, none of the studied socio-demographic characteristics had shown any significant association with uptake of first IPTp dose. By contrast, in Bangui, Central Africa, pregnant women with at least a secondary school education, were twice more likely to comply with IPTp compared to those with lower level of education, while those with some form of income, were four times more likely to comply with IPTp compared to those with no income at all [37]. Another study in Ibadan, Nigeria, showed that educational attainment of nine years or less, significantly predicted IPTp non-usage [36].

Out of all the variables studied, only knowledge predicted uptake of first IPTp dose, which is similar to findings of a previous study in a rural community, where those with knowledge of IPTp were more than twice more likely to take IPTp compared to those who had no knowledge of it. Similarly, the same study found no significant association between educational level, age group, and occupation, with IPTp uptake [46]. Unlike sleeping under an insecticide-treated net (ITN) [64], motivation and self-efficacy played no role in uptake of first IPTp dose, rendering IPTp the intervention with the greatest potential for achieving universal coverage if properly implemented. This is because taking IPTp is a one-time activity, unlike sleeping under an ITN which is a daily activity, requiring some form of motivation. Giving directly-observed treatment (DOT) also eliminates the chances of forgetfulness.

Among the limitations of the study was the subjective nature of some of its questions, and as such, the findings should be interpreted within that context. The study design also has an inherent problem of temporal relationship, making it difficult to ascertain whether they ab initio had a high knowledge of IPTp, which prompted them to take their first IPTp doses, or whether they got to have a high knowledge of IPTp only after they had taken it. Longitudinal studies are thus recommended to eliminate this problem of temporal relationship associated with cross-sectional studies. Important factors like ability to purchase the medication, should be incorporated in future studies. In addition, whether they had purchased the medication but not taken it needs to be studied. It is also recommended that to get more insight into the problem of IPTp uptake, especially with regards to motivation and self-efficacy, future studies should incorporate focus group discussions with patients and key informant interviews with antenatal staff and other service providers. Efforts should also be made to provide the drug free of charge at health centres, while observing DOT.

## Conclusion

Knowledge and uptake of IPTp were below optimal levels in this study. Since knowledge of IPTp significantly predicts uptake of first IPTp dose, there is the need to implement health education campaigns to raise the awareness of pregnant women and their families on the need to receive and comply with it. IPTp should also be strongly emphasised at antenatal clinics during health education sessions.

## Supplementary Information

**Additional file 1.** Questionnaire This questionnaire contains five sections, thus: respondents’ characteristics; knowledge; motivation; self-efficacy and practice.

**Additional file 2.** Data set This data set on IPTp coverage among antenatal care attendees of the health facility is presented in a Microsoft Excel spreadsheet. Information for each respondent is presented in one row.

## Data Availability

All data generated or analysed during this study are included as additional supporting files.

## Abbreviations

ANC: Antenatal care
DOT: Directly-observed treatment
ITN: Insecticide-treated net
IPTp: Intermittent preventive treatment in pregnancy
FMOH: Federal Ministry of Health
PHC: Primary Health Care
SP: Sulphadoxine-Pyrimethamine
WHO: World Health Organization
