# Supplementary material for: Determinants of uptake of first dose of intermittent preventive treatment among pregnant women in a secondary health Centre in Maiduguri, Nigeria
Source: BMC Pregnancy Childbirth. 2020 Nov 25;20:726. doi: 10.1186/s12884-020-03388-8 (PMC7687802; doi:10.1186/s12884-020-03388-8)
Supplement: Supplementary file 1 — Additional file 1. Questionnaire This questionnaire contains five sections, thus: respondents’ characteristics; knowledge; motivation; self-efficacy and practice. [file 12884_2020_3388_MOESM1_ESM.docx]

**JERIN TAMBAYOYI**

**Umarni:** Don Allah, a amsa dukkan tambayoyi, kuma a alamta (√) amsa guda daya kawai ga kowane kalamia ɗan akwatin da ya dace.

**Matashiya:** Don Allah, a ƙoƙarta a amsa dukkan tambayoyin tsakani da Allah domin wannan ba jarrabawa ba ce ko gwaji.

**SASHEN i**

**Umarni:** Don Allah a alamta (√) amsar da ta fi dacewa ko a cike amsar da kalamai.

1. Shekaru: ………….

2. Tsarin iyali

- Auren tilo
- Auren mata fiye da ɗaya

3. Matsayin Ilimi

- Ban yi makaranta ba
- Na yi Firamare
- Na yi sakandare
- Na yi na gaba da sakandare

4. Matsayin aiki

- Babu/matar aure
- Ina aikin kai na
- Aikin gwamnati
- Aikin masana’anta
- Ɗalibta

5. Kuɗin da nake samu duk wata Naira….……………….

6. Zama a Maiduguri

- Mazaunin din-din-din
- Gudun hijira

7. Adadin goyon ciki (har da wannan)………………….

8. Kin taɓa yin ɓari?

- Eh
- A’a

9. Tsawon wane lokaci ne ba ki ga watan al’adarkiba? Adadin wata……………………………………..

**SASHEN ii**

**Umarni:** Don Allah a alamta haka ‘√’ ga amsar da ta dace daga cikin zaɓin da aka ba da, wato, kodai **EH, A’A**, ko **BAN SANI BA** ga kowane ɗaya daga kalaman da ke tafe a ƙasa

| **Lamba** | | **Tambayoyi** | **Amsoshi** | | |
| --- | --- | --- | --- | --- | --- |
|  |  | | **EH** | **A’A** | **BAN SANI BA** |
| 1. | Kina da masaniya akan maganin da ake bayarwa na kariya lokacin goyon ciki? | |  |  |  |
| 2. | **Wane irin magani ake bayarwa don kariya daga cutar malariya lokacin goyon ciki?** | | | | |
|  | Chloroquine | |  |  |  |
|  | Fansidar | |  |  |  |
| 3. | **Nawa ne adadin kwayoyin maganin kariya daga cutar malariya da ake bayarwa kowane lokaci ga mai goyon ciki?** | | | | |
|  | Ƙwaya 2 | |  |  |  |
|  | Ƙwaya 3 | |  |  |  |
|  | Ƙwaya 4 | |  |  |  |
| 4. | Maganin da ake ba wa masu goyon ciki don kariya daga cutar malariya zai iya zama mai illa akan cikin da nake goyo | |  |  |  |
| 5. | Ana iya shan maganin kariya daga cutar malariya ba tare da an ci abinci ba? | |  |  |  |

**SASHEN iii**

**Umarni:** Don Allah a alamta (√), amma amsa ɗaya ake bukata ga kowane kalami.

Kowane kalami za a amsa ne daidai da sikili mai hawa 5 wadda ya fara daga

1 = Ba kyau sosai, zuwa 5 = Da kyau sosai; DA KUMA

1 = Ba daɗi sosai, zuwa 5 = Da daɗi sosai.

| **a** | **Don Allah a gaya mana yaya kyaun ko rashin kyaun waɗannan game da lafiyarki.** | **Ba kyau sosai** | **Haka dai ba kyau** | **Ba kyau ba daɗi** | **Haka dai da kyau** | **Da kyau sosai** |
| --- | --- | --- | --- | --- | --- | --- |
| 1. | Rinƙa shan maganin kariya daga cutar malariya da aka ba ni yayin goyon ciki |  |  |  |  |  |
| 2. | Rinƙa shan dukkan magungunan kariya daga cutar malariya da aka bani ko da ina jin lafiyata ƙalau |  |  |  |  |  |
| **b** | **Don Allah a gaya mana yaya daɗi ko rashin daɗin waɗannan halayen a gareki.** | **Ba dadi sosai** | **Haka dai ba dadi** | **Ba kyau ba daɗi** | **Haka dai da dadi** | **Da dadi sosai** |
| 3. | Rinƙa shan maganin kariya daga cutar malariya da aka ba ni yayin goyon ciki |  |  |  |  |  |
| 4. | Rinƙa shan dukkan magungunan kariya daga cutar malariya da aka bani ko da ina jin lafiyata ƙalau |  |  |  |  |  |

**Umarni:** Don Allah a alamta (√), amma amsa ɗaya ake bukata ga kowane kalami.

Kowane kalami za a amsa ne daidai da sikili mai ma’auni shida:

1: Ƙarya ne sosai

2: Akasari ƙarya ne

3: Ƙarya ne

4: Gaskiya ne

5: Akasari gaskiya ne

6: Gaskiya ne sosai

|  | **Don Allah a gaya mana yaya gaskiya ko rashin gaskiyar ta ke a gareki** | **Ƙarya ne sosai** | **Akasari ƙarya ne** | **Ƙarya ne** | **Gaskiya ne** | **Akasari gaskiya ne** | **Gaskiya ne sosai** |
| --- | --- | --- | --- | --- | --- | --- | --- |
|  | Mutanen da ke da muhimmanci a gare ki suna tsammanin yakamata ki… |  |  |  |  |  |  |
| 5. | Rinƙa shan maganin kariya daga cutar malariya da aka ba ki yayin goyon ciki |  |  |  |  |  |  |
| 6. | Rinƙa shan maganin kariya daga cutar malariya da aka baki ko da kina jin lafiyarki ƙalau |  |  |  |  |  |  |

**SASHEN iv**

**Umarni:** A wannan sashe ana tambaya akan kimar wahala ko sauƙi da iyawa ko rashin iya gudanar da wasu ayyuka

Don Allah a alamta (√), amma amsa ɗaya ake bukata ga kowane kalami. Kowane kalami za a amsa ne daidai da sikili mai ma’auni huɗu.

| **a.** | **A halin yanzu yaya wahalar ko sauƙin yadda zaki iya….** | **Da wahala sosai** | **Da wahala** | **Da sauƙi** | **Da sauƙi sosai** |
| --- | --- | --- | --- | --- | --- |
|  |  | **1** | **2** | **3** | **4** |
| 1. | Shanye dukkan maganin kariya daga zazzaɓin cizon sauro lokacin goyon ciki? |  |  |  |  |
| 2. | Shanye dukkan maganin kariya daga zazzaɓin cizon sauro ko da kikan ji ba daɗi? |  |  |  |  |

**SASHEN v**

54. Yanzu da kike ɗauke da wannan cikin kin sha maganin kariya daga zazzabin cizon sauro yayin goyon ciki?

- Eh
- A’a

An Gode da haɗin kai da aka ba da!

**QUESTIONNAIRE**

**Instructions:** Please answer ALL questions and please TICK (√) only one answer for each statement, in the appropriate box.

**Reminder:** Please try to answer all questions honestly as this is not an examination or a test.

**SECTION i**

**Instructions:** Please tick (√) the most appropriate option or fill in your response.

1. Age: …….... years

2. Family type

- Monogamy
- Polygamy

3. Educational status

- No formal educational
- Primary
- Secondary
- Tertiary

4. Occupation

- None/housewife
- Self-employed
- Government employed
- Private employment
- Student

5. Monthly income in naira ……………

6. Residence in Maiduguri

- Permanent Resident
- Internally displaced person

7. Total number of pregnancies (including this pregnancy)………….

8. Do you have any previous history of miscarriage?

- Yes
- No

9. For how long have you not seen your menses?.......................(months)

**SECTION ii**

**Instructions:** Please tick (√) the most appropriate option based on the options provided which are: **YES**, **NO** or **I DON’T KNOW** for each of the statements below.

| **S/NO** | **Questions** | **Response** | | |
| --- | --- | --- | --- | --- |
|  |  | **YES** | **NO** | **I DON’T KNOW** |
| 1. | Are you aware of the medicine given during pregnancy for protection against malaria? |  |  |  |
| 2. | **Which medicine is given for protection against malaria during pregnancy?** | | | |
|  | Chloroquine |  |  |  |
|  | Fansidar |  |  |  |
| 3. | **How many tablets of the medicine for protection against malaria during pregnancy should be given at each time?** | | | |
|  | 2 tablets |  |  |  |
|  | 3 tablets |  |  |  |
|  | 1. tablets |  |  |  |
| 4. | The medicine given to pregnant women for protection against malaria during pregnancy can be harmful to the pregnancy |  |  |  |
| 5. | Can the medicine given for protection against malaria in pregnancy be taken on an empty stomach? |  |  |  |

**SECTION iii**

**Instructions:** Please TICK (√) only one answer for each statement. Each statement is answered based on a 5-point scale which ranges from 1 = very bad to 5 = very good; or from 1 = very unpleasant to 5 = very pleasant.

| a | **Please tell us how good or bad the following are for your health** | **very bad** | **somewhat bad** | **neither bad nor good** | **somewhat good** | **very good** |
| --- | --- | --- | --- | --- | --- | --- |
|  | For the remaining duration of your pregnancy, how good or bad would it be for your health…. |  |  |  |  |  |
| 1. | To take all the medicines given to me for preventive treatment of malaria in pregnancy? |  |  |  |  |  |
| 2. | Take all the medicines given to me for preventive treatment of malaria in pregnancy even when I don’t feel sick |  |  |  |  |  |
| **b** | **Please tell us how pleasant or unpleasant it would be for you to do the behaviour** | **very unpleasant** | **somewhat pleasant** | **neither unpleasant nor pleasant** | **somewhat pleasant** | **very pleasant** |
|  | For the remaining duration of your pregnancy, how pleasant or unpleasant would it be for you…. |  |  |  |  |  |
| 3. | To take all the medicines given to me for preventive treatment of malaria in pregnancy? |  |  |  |  |  |
| 4. | To take all the medicines given to me for preventive treatment of malaria in pregnancy even when I don’t feel sick |  |  |  |  |  |

**Instructions:** Please TICK (√) only one answer for each statement. Each statement is answered based on a 6-point scale which is:

1: Very untrue

2: Mostly untrue

3: Untrue

4: True

5: Mostly true

6: Very true

|  | **Please tell us how true or untrue it is for you** | **very untrue** | **mostly untrue** | **untrue** | **true** | **mostly true** | **very true** |
| --- | --- | --- | --- | --- | --- | --- | --- |
|  | Most people who are important to me think I should… |  |  |  |  |  |  |
| 5. | Take all the medicines given to me for preventive treatment of malaria in pregnancy? |  |  |  |  |  |  |
| 6. | Take all the medicines given to me for preventive treatment of malaria in pregnancy even when I don’t feel sick |  |  |  |  |  |  |

**SECTION iv**

**Instructions:** This section asks about level of difficulty/ease and effectiveness/ineffectiveness in performing certain tasks.

Please TICK (√) only one answer for each statement. Each statement is answered according to a four point scale which is:

1: Very hard

2: Hard

3: Easy

4: Very easy

| **a** | **Right now, how easy or hard would it be for you to…** | **Very hard**  **1** | **Hard**  **2** | **Easy**  **3** | **Very easy**  **4** |
| --- | --- | --- | --- | --- | --- |
| 1. | Take all the medicines given to you for prevention against malaria during pregnancy? |  |  |  |  |
| 2. | Take all the medicines given to you for prevention against malaria during pregnancy even when you experience mild discomfort taking them? |  |  |  |  |

**SECTION v**

In this pregnancy, have you taken the medicines given for protection against malaria during pregnancy?

- Yes
- No

Thank you for your co-operation!
